# Comprehensive Diagnosis of Viral Hepatitis in Spain: Bases for Implementation

**DOI:** 10.3390/v17050667

**Published:** 2025-05-03

**Authors:** Joaquin Cabezas, Antonio Aguilera, Federico García, Raquel Domínguez-Hernández, Araceli Casado-Gómez, Nataly Espinoza-Cámac, Miguel Ángel Casado, Javier Crespo

**Affiliations:** 1 Gastroenterology and Hepatology Service, Marqués de Valdecilla University Hospital, 39008 Santander, Spain; javiercrespo1991@gmail.com; 2 Clinical and Translational Research Group in Digestive Diseases, Valdecilla Research Institute (IDIVAL), 39011 Santander, Spain; 3 Microbiology Service and Instituto de Investigación Sanitaria de Santiago (IDIS), University Clinical Hospital of Santiago de Compostela, 15706 A Coruña, Spain; 4 Microbiology Department, University of Santiago de Compostela, 15705 A Coruña, Spain; 5 Microbiology Service, San Cecilio Clinical University Hospital, 18007 Granada, Spain; fegarcia@ugr.es; 6 Instituto de Investigación Biosanitaria Ibs.GRANADA, 18012 Granada, Spain; 7 Centro de Investigación Biomédica en Red de Enfermedades Infecciosas (CIBERINFEC), 28029 Madrid, Spain; 8 Pharmacoeconomics & Outcomes Research Iberia (PORIB), 28224 Madrid, Spain; 9 Faculty of Medicine, University of Cantabria, 39011 Santander, Spain

**Keywords:** hepatitis B, hepatitis C, hepatitis D, integrated diagnosis, point-of-care testing

## Abstract

In 2022, scientific societies agreed on a document with recommendations for a comprehensive diagnosis of viral hepatitis (B, C, and D). The aim was to evaluate the situation in Spain regarding the comprehensive diagnosis of viral hepatitis in a single blood draw before it is recommended. A panel of experts prepared a structured survey directed at hospitals (public or private with teaching accreditation) with ≥200 beds (sent 20 October 2022, closed 1 December 2022). The response rate was 61% (79/129; 52 hospitals with >500 beds). Among the participating hospitals, all could perform tests for HBsAg, anti-HCV, and HIV serology; 94% could perform PCR testing for HCV, 63% could test for anti-HDV, and 28% could test for HDV-RNA (67% [53/79] outsourced this testing). Point-of-care (POC) testing availability was low (24%), with 84% of these tests being supervised by the reference microbiological laboratory and the results being registered in the patients’ medical history. Ninety percent of the centers carried out the diagnosis in a single step (99% HCV, 70% HBV, 48% HDV, and 44% HBV-HDV). In addition, 77% used some communication strategy when an active infection was encountered (100% HCV, 49% HBV, and 31% HDV). Only 20% had an automated system for scheduling a specialist physician appointment. Most hospitals had the means for a comprehensive diagnosis of viral hepatitis in a single sample, but <50% could test for HBV/HDV. Alerts for continuity of care were available for HCV, but not HBV or HDV. POC device implementation is important for decentralized testing.

## 1. Introduction

Viral hepatitis is an infectious disease that results in high morbidity and mortality due to liver damage [1,2]. Hepatitis B, C, and D can evolve with a chronic and progressive course and the development of long-term clinical complications, such as cirrhosis, liver decompensation, hepatocellular carcinoma, and the need for liver transplantation or death [1]. Hepatitis A manifests as an acute course and complete resolution in most cases [1]. Worldwide, more than 1.5 million new infections occur each year, mainly due to hepatitis B virus (HBV) and hepatitis C virus (HCV) infection [1]. In addition, mortality due to hepatitis continues to be high, which is why screening and diagnosis are important for early and timely detection and treatment [3,4]. The treatments currently available can achieve the control or cure of these infections [5,6]. However, in Spain, one-quarter of people with chronic hepatitis B or C visit specialist physicians late in their disease course and thus have advanced liver disease [7]. The presence of HBV is required for the hepatitis D virus (HDV) to replicate, and chronic HDV infection is currently the viral liver disease with the highest risk of progression [8]. Regarding human immunodeficiency virus (HIV), existing therapeutic alternatives can reduce the number of infections, although high numbers of late diagnoses continue to be reported (47.6% in 2018) [9,10]. On the other hand, comorbid HIV infection and viral hepatitis often leads to a more rapid progression of viral hepatitis [11,12,13].

The epidemiological magnitude of viral hepatitis and HIV led the World Health Organization (WHO) to target their elimination along with sexually transmitted infections (STIs) by 2030 [11]. Consequently, many countries have developed public health strategies at the global level to achieve this goal. In line with this, in 2022, the document “Recommendations for the implementation of a comprehensive diagnosis of viral hepatitis” was drafted in Spain, endorsed by the following scientific societies: the Spanish Association for the Study of the Liver (AEEH), the Alliance for the Elimination of Viral Hepatitis in Spain (AEHVE), the Study Group of Viral Hepatitis (GEHEP), the Spanish Society of Infectious Diseases and Clinical Microbiology (SEIMC), and the Spanish Society of Digestive Pathology (SEPD) [14]. The document establishes a series of recommendations to carry out a comprehensive diagnosis of hepatitis B, C, and D, as well as the incorporation of screening for hepatitis A virus (HAV) and HIV in the same blood sample, with the purpose of, in the event of a positive serological result, screening for the other viruses, including determining the viral load if necessary [6,15]. In addition, this comprehensive diagnosis should be accompanied by effective diagnostic simplification strategies such as reflex testing, the incorporation of informative alerts, the decentralization of diagnosis, and the implementation of screening programs. A comprehensive diagnosis together with other strategies would help increase the number of cases detected, reduce the time to access treatment, and minimize patient loss to follow-up, thus achieving the objectives set forth by the WHO [12,14].

We do not have information on the status of the comprehensive diagnosis in Spain prior to the dissemination of the document in question [16]. We only have the results of a previous survey carried out in Spanish hospitals where the available diagnostic resources and the process of diagnosing HCV infection were addressed. To assess the impact of new strategies, it is necessary to know the status prior to implementation. For this reason, the aim of this manuscript is to describe the status of a comprehensive diagnosis of viral hepatitis and HIV before the publication of the document on the recommendations for hospitals in Spain, as well as the status of reflex testing implementation.

## 2. Materials and Methods

**Figure 1:**
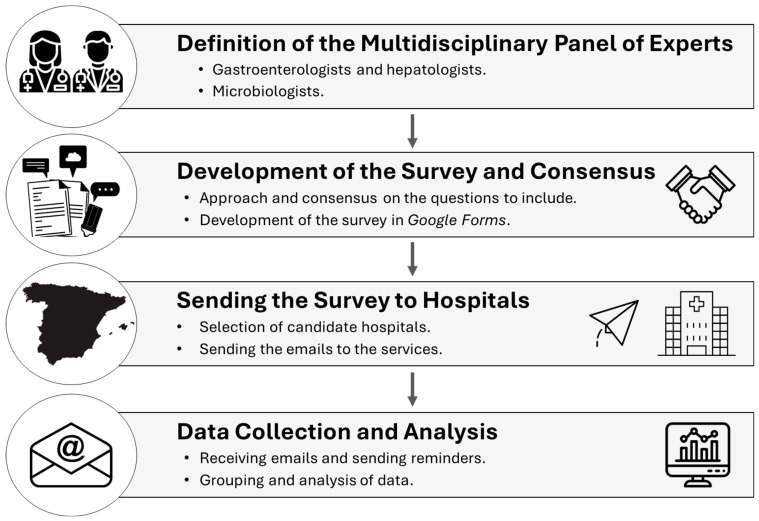
Outline of the methodology.

A descriptive cross-sectional study was designed to evaluate the status of a comprehensive diagnosis of viral hepatitis. A structured survey was developed to collect data from Spanish hospitals in the context of the diagnosis of viral hepatitis before the implementation and publication of the recommendation document [14]. A panel of experts made up of microbiologists, hepatologists, and digestives drafted and agreed on the list of questions included in the survey. The survey was designed via the Google Forms platform. The survey was sent to the person responsible for the diagnosis of viral hepatitis in the selected hospitals. The hospitals selected included public and private hospitals with more than 200 beds listed in the National Catalogue of Hospitals (NCHs) [17]. The invitation to participate in the survey, which included the link, was sent by email on 20 October 2022. Reminders were sent before the first deadline set for the receipt of results (1 December 2022). The final deadline for data receipt was 28 February 2023 (Figure 1).

### Survey and Collection Parameters

The information collected in the survey was grouped into the following sections: (1) respondent and hospital center information, (2) diagnosis of hepatitis B and D in the center, (3) availability of reflex testing and typology, (4) comprehensive diagnosis including HAV and HIV, (5) availability of point-of-care (POC) testing, (6) integration of screening programs, (7) implementation of alerts and continuity of care, and (8) opinion questions (Supplementary Materials). Consent for participation in the project was requested before completing the survey.

Regarding the diagnosis of hepatitis B and D, the methods of detecting antibodies and/or determining viral load were queried. The questions regarding the availability of reflex testing were focused on screening tests that are routinely implemented in clinical practice at each center, rather than on theoretical or technical laboratory capacity. Depending on the type of hepatitis, this included the systematic use of reflex testing for viral load determination. For comprehensive diagnosis, four reflex testing subtypes (HBV, HDV, dual HBV-HDV, and HCV reflex testing) were categorized according to the systematic screening of the same analytical sample (Table 1). In addition, information was requested on how HAV and HIV screening was performed.

Similarly, information was requested on the use of POC testing, defined as the set of tests available at the place of patient care involving obtaining a blood sample for a rapid diagnosis, as well as the type of test, supervision by the central microbiology laboratory, and subsequent registration of the result in patient medical records. The types of POC tests used included the rapid capillary blood antibody test, the HCV antibody test in capillary blood or saliva (Oraquick^®^ HCV), the GeneXpert^®^ HCV test (polymerase chain reaction, PCR), and the Died Blood Sample (DBS).

In addition, the questions about the integration of the screening programs were related to the availability of a regional plan for the management of viral hepatitis, systematic screening in addiction centers, and the type of test administered to treat patients at risk of reinfection. Furthermore, as part of the strategies of communicating results within the hospital center and the continuity of care in cases of evidence of active viral hepatitis, information on the availability and type of alerts to a specialist physician about what type of hepatitis was implemented, as well as if there was an automated system for scheduling an appointment with a specialist physician, was requested.

Finally, the opinion questions were aimed at determining whether there is a need to analyze HAV and HIV together with the other hepatitis viruses, as in patients with suspected STIs.

The statistical analysis included the calculation of absolute and relative frequencies in percentages, since only qualitative variables were evaluated in this descriptive study.

## 3. Results

### 3.1. Respondent and Hospital Center Information

A total of 129 hospitals were contacted, resulting in a response rate of 61% (*n* = 79). Among all participating centers, 97% were university teaching hospitals. In most cases, the specialty of the respondent was microbiology (99%). According to the number of hospital beds, 27 hospitals from Group 2 (200–500 beds), 37 from Group 3 (501–1000 beds), and 15 hospitals from Group 4 (>1000 beds) participated. A summary of the results is shown in Table 2.

### 3.2. Reflex Testing of HBV, HCV, and HDV

(a) HBV. All the participating hospitals had the ability to perform HBV serological analyses. Reflex testing of HBV was performed in 70% of the hospitals (*n* = 50). In patients with chronic HBV infection, HAV testing was performed in 25% (*n* = 20) of the centers, and of these, 75% (*n* = 15) of tests were performed on the same sample. Similarly, HIV testing was performed for only 33% (*n* = 26) of chronic hepatitis B patients, and it was also performed mostly on the same sample.

(b) HCV. Reflex testing of HCV was performed in all the participating hospitals except one (99%; *n* = 70). HAV testing was performed for 20% (*n* = 16) of patients with chronic hepatitis C, and of these, 75% (*n* = 12) of tests were carried out on the same sample. Similarly, HIV testing was performed for only 38% (*n* = 30) of chronic hepatitis C patients, and it was also performed mostly on the same sample.

(c) HDV. Regarding the diagnosis of hepatitis D, 63% (*n* = 50) of the participating centers performed tests for anti-HDV detection and 28% (*n* = 22) performed tests for HDV-RNA detection. Among the centers that did not perform tests for HDV-RNA detection, 93% (*n* = 53) outsourced these tests to another center. Reflex testing of HDV (48%; *n* = 34) and dual HDV-HBV were performed in 31 centers (44%).

### 3.3. POC Testing, Integration of Screening Programs and Continuity of Care

POC rapid diagnostic tests were available only in 24% (*n* = 19) of the surveyed hospitals. Among the hospitals that performed these tests (*n* = 19), the GeneXpert^®^ HCV test was the test most commonly used (58%; *n* = 11), followed by the DBS (47%; *n* = 9), the Rapid Capillary Blood Antibody test (32%; *n* = 6), and the Oraquick^®^ HCV test (5%; *n* = 1). In addition, the reference microbiology laboratories monitored 84% (*n* = 16) of the POC results, and 95% (*n* = 18) registered these results in the patients’ medical records.

Regarding the integration of the screening programs, 77% (*n* = 61) of the centers reported having a regional plan for the management and/or elimination of viral hepatitis; 71% (*n* = 56) implemented systematic screening in addiction centers, the most widely used method being conventional extraction (*n* = 68), followed by the DBS (*n* = 18) and the GeneXpert^®^ test (*n* = 9).

In cases of evidence of active viral hepatitis, 77% (*n* = 61) of the centers had some type of system to alert the specialist physician, with 100% (*n* = 61), 49% (*n* = 30), and 31% (*n* = 19) of the centers reporting evidence of hepatitis C, B, and D, respectively. In the cases of positive serology, only 20% (*n* = 16) of the centers had an automated system for scheduling an appointment with a specialist physician, and the service in charge of managing this system was reported in most cases.

### 3.4. Comprehensive Diagnosis Needs and Qualitative Information

For patients with positive serology for hepatitis B and C, 68% (*n* = 54) of those surveyed thought that HAV testing should be performed on the same blood sample, and 91% (*n* = 72) thought that supplemental HIV testing should also be performed. The arguments for not including hepatitis A testing were that there were differences in hepatitis A, B, and C epidemiology, that they did not share the same transmission or contagion mechanism; and that the patient profiles were different. The arguments for not determining HIV infection status in patients with positive serology for hepatitis B or C were the possible legal implications of the inclusion of the test, that there should be regulations, and that the test should be performed only in the population at risk. One of the limitations noted was that there is often not enough sample (serum or plasma) for all of the diagnostic tests to be performed; therefore, it is necessary to request a new sample and ask the doctor for an extension of the deadline for results.

In the case of suspected STIs, 96% (*n* = 76) of the respondents thought that comprehensive testing for viral hepatitis should be carried out, considering mostly (*n* = 75) that a blood sample should be available from these patients, and 99% (*n* = 78) thought that they should also be tested for HIV infection.

## 4. Discussion

The status of viral hepatitis diagnosis as well as referral and awareness of positive cases, among others, prior to the implementation of a strategy, is essential to be able to assess future impact. The results of this survey reveal a 90% adoption rate of reflex testing in hospital settings. Reflex testing of HCV is particularly noteworthy, as it was performed in almost all of the centers surveyed (99%). Reflex testing of HBV was the second most common test, although it has yet to be implemented in more than one-quarter of hospitals. On the other hand, both reflex testing HDV and the dual HBV-HDV were less common, performed in fewer than 50% of the surveyed hospitals. These data could be influenced by the high percentage of hospitals that outsource HDV-RNA detection (93%), highlighting the potential underdiagnosis of HDV, as indicated by previous studies [18]. This aspect is crucial since the routine detection of anti-HDV antibodies among HBV surface antigen (HBsAg) carriers has been shown to significantly increase the number of diagnoses of HDV infection [19,20]. However, the low overall prevalence of HDV infection in individuals who are carriers of HBsAg must also be considered [21,22,23]. Similarly, our findings reveal areas of substantial improvement in terms of alerts to specialist physicians, which are predominantly available for hepatitis C (100%) but notoriously absent for other forms of hepatitis. Similarly, an automated system for scheduling an appointment with a specialist physician was present in only 20% of hospitals, despite its proven effectiveness in improving continuity of care and its benefit in terms of cost effectiveness [24].

Regarding diagnosis through POC testing, only one-quarter of hospitals (24%; 19 of 79) had integrated this modality. Given the importance of bringing the diagnosis closer to the populations at greatest risk, it is imperative to continue promoting the decentralization of testing. On the other hand, the monitoring of results and their registration into patients’ medical records were well established in the participating hospitals.

On the other hand, the high prevalence of viral hepatitis among STI patients is remarkable [25]. Despite the widespread belief among experts about the need for a comprehensive diagnosis of viral hepatitis in the event of suspected STIs, a Spanish study showed that 38% of patients with syphilis lacked HCV serology, whereas 90% of patients with HIV did. The latter data are consistent with the results of our survey and highlight the disparity in detection between different STIs. The WHO has outlined targets for the elimination of viral hepatitis, HIV, and STIs, which represents a significant challenge for public health [11]. In addition, the strategies recommended by various agencies highlight the importance of joint interventions focused on the screening, diagnosis, and treatment of these viral infections and STIs [11,13,26]. Particular emphasis should be placed on addressing the needs of key populations, such as men who have sex with men, transgender people, sex workers, people who inject drugs, and people in prison settings [27]. Many countries have already implemented national strategies for STIs, which would facilitate the incorporation of tests for viral hepatitis [28]. A recent study that sought to increase the comprehensive diagnosis of HIV, HBV, HCV, and STIs through tests based on indicator conditions reported high rates of positivity, especially for HCV, and a considerable increase in the number of tests performed [29]. The integration of these tests not only improves acceptance by patients but also generates financial savings [11,30]. One study showed that testing for HBV in unvaccinated adults seeking care for STIs could prevent new infections and reduce costs to the health care system [31].

Finally, the results of this study showed that the determination of hepatitis A in the positive case of HBV or HCV was infrequent. Determining the state of immunity against HAV in patients with chronic hepatitis B or C is crucial for assessing the need for vaccination in populations at risk [32]. Although coinfection is rare outside of epidemic outbreaks associated with sexual transmission, men who have sex with men, and other risk groups, such as people with STIs, the data collected reflect opinions that advocate the consideration of coinfection. In addition, the recommendations for comprehensive diagnosis also serve to implement primary prevention measures (vaccines) against HBV in susceptible patients, as long as the recommendations in this regard are not universal.

Among the limitations of the study, the relatively low response rate of the centers surveyed stands out (61%), although this is within the range observed in similar studies (56% [16] and 80% [33]). As is common in self-report studies, there is an inherent risk of bias due to the personal perceptions of the respondents. Furthermore, the lack of quantitative variables limits the extent of statistical analysis that can be performed.

The implementation of a comprehensive diagnosis of viral hepatitis and HIV through a single blood draw could significantly increase early detection rates, reduce the time to treatment, and minimize patient loss to follow-up, thus helping achieve the elimination targets set by the WHO. Our results show the situation prior to the recommendations but, to evaluate any changes in the implementation of a comprehensive diagnosis of viral hepatitis in hospital settings, future monitoring will be necessary. Therefore, our future efforts should focus on identifying barriers to the implementation of these recommendations in our hospitals, evaluating the economic impact of this implementation, and comparing the effectiveness of different strategies for communicating results and automated systems to improve continuity of care.

In summary, a comprehensive diagnosis of viral hepatitis and HIV is key to strengthen the control of these infections. The results of this survey underscore the opportunity to implement comprehensive viral hepatitis diagnostic techniques and philosophies in hospital settings as well as promote further dissemination and training on key recommendations, similar to the initial implementation of reflex testing, in forums of relevant interest. The experience gained with hepatitis C should serve as a model for the implementation and optimization of plans for the diagnosis and treatment of other types of viral hepatitis, especially the management of HDV, taking advantage of the diagnosis as a strategic tool. In addition, promoting and ensuring continuity of care through effective alerts are essential steps towards eliminating these infections. Our next efforts should focus on expanding the implementation of comprehensive diagnostics, optimizing the use of POC (reflex testing) devices, to maximize the impact on the diagnosis and treatment of viral hepatitis and HIV.

## Figures and Tables

**Table 1:** Categorization of the type of reflex testing.

Reflex Testing Type	Systematic Determination of the Same Analytical Sample
HBV	Systematic determination of HBV-DNA in the same analytical sample when HBsAg is first detected in the patient.
HDV	Systematic determination of antibodies against HDV (anti-HDV) in the same analytical sample when HBsAg is first detected in the patient.
Dual HBV-HDV	Systematic determination of HDV antibodies (anti-HDV) when HBsAg is first detected in the patient, together with the systematic determination of HDV-RNA in the same sample in all patients in whom anti-HDV is detected for the first time.
HCV	Systematic determination of HCV by PCR in all patients in whom anti-HCV is detected for the first time in the same sample.

Abbreviations: HBV, hepatitis B virus; HCV, hepatitis C virus; HDV, hepatitis D virus.

**Table 2:** Summary of the results obtained in the survey.

Variables	Response Alternatives	*n*	%
**Consent to Participate in the Project (*n* = 129)**			
Guest centers	Total	129	100%
Consent	No	50	39%
	Yes	79	61%
**1.** **Respondent and Center Data (*n* = 79)**			
Specialty	Microbiology	78	99%
	Infectious Diseases	1	1%
Number of hospital beds	Group 2 (200–500 beds)	27	34%
	Group 3 (501–1000 beds)	37	47%
	Group 4 (>1000 beds)	15	19%
Reference population (number of inhabitants)	Minimum	16,000	
	Maximum	2,000,000	
University teaching hospital	No	2	3%
	Yes	77	97%
**2.** **Diagnosis of Hepatitis D (*n* = 79)**			
Determinations of anti-HDV in the center	No	29	37%
	Yes	50	63%
*HDV-RNA determination in the center*	No	57	72%
	*No, because it is outsourced*	*53*	*93%*
	*No, it is not outsourced either*	*4*	*7%*
	Yes	22	28%
**3.** **Reflex Testing of Viral Hepatitis (*n* = 79)**			
Reflex testing is performed	No	8	10%
	Yes	71	90%
*Type of reflex testing (n = 71)*	*HCV*	*70*	*99%*
	*HBV*	*50*	*70%*
	*HDV*	*34*	*48%*
	*Dual HBV-HDV*	*31*	*44%*
**4.** **Other Diagnostic Recommendations (*n* = 79)**			
**Hepatitis A**			
In patients with chronic hepatitis B, the level of anti-HAV IgG or total IgG was determined	No	59	75%
	Yes	20	25%
	*In the same sample*	*15*	*75%*
	*In another sample*	*5*	*25%*
In patients with chronic hepatitis C, anti-HAV IgG or total IgG is determined	No	53	67%
	Yes	16	20%
	*In the same sample*	*12*	*75%*
	*In another sample*	*4*	*25%*
**Human Immunodeficiency Virus (HIV)**			
In patients with chronic hepatitis B, anti-HIV is determined	No	53	67%
	Yes	26	33%
	*In the same sample*	*19*	*73%*
	*In another sample*	*7*	*27%*
In patients with chronic hepatitis C, anti-HIV is determined	No	49	62%
	Yes	30	38%
	*In the same sample*	*19*	*63%*
	*In another sample*	*11*	*37%*
**5.** **General Measures: Point-of-Care (*n* = 79)**			
Point-of-Care (POC) tests	No	60	76%
	Yes	19	24%
*Type of tests in the POC (n = 19)*	*GeneXpert^®^ HCV*	*11*	*58%*
	*Dried Blood Sample*	*9*	*47%*
	*Rapid capillary blood antibody test*	*6*	*32%*
	*Oraquick^®^ HCV (capillary blood or saliva)*	*1*	*5%*
*Central Microbiology laboratories monitor POC results (n = 19)*	*No*	*3*	*16%*
	*Yes*	*16*	*84%*
*POC results integrated in the clinical history* *(n = 19)*	*No*	*1*	*5%*
	*Yes*	*18*	*95%*
**6.** **Integration of Screening Programs (Implementation of Viral Hepatitis Elimination Programs) (*n* = 79)**			
Community or regional plan	No	18	23%
	Yes	61	77%
Systematic screening in addiction centers	No	23	29%
	Yes	56	71%
Screening in previously treated patients at risk of reinfection	Conventional extraction	68	86%
	Dried Blood Sample	18	23%
	GeneXpert^®^	9	11%
**7.** **Centers’ Result Communication Strategies (*n* = 79)**			
The specialist physician is alerted on the existence of active viral hepatitis (alerts for the following types: HBV, HDV, or HCV)	No	18	23%
	Yes	61	77%
	*HBV*	*30*	*49%*
	*HDV*	*19*	*31%*
	*HCV*	*61*	*100%*
Automated system for scheduling an appointment with a specialist physician for patients with active infection	No	63	80%
	Yes	16	20%
	*Yes, alert to appointment management*	*15*	*94%*
	*No, alert to appointment management*	*1*	*6%*
**8.** **Opinion Questions (*n* = 79)**			
If HBV+ or HCV+, HAV screening should be performed on the same blood sample	No	25	32%
	Yes	54	68%
If HBV+ or HCV+, HIV screening should be performed on the same blood sample	No	7	9%
	Yes	72	91%
If STIs are suspected, a comprehensive screen for viral hepatitis should be performed	No	3	4%
	Yes	76	96%
	*Yes, a blood sample must be available*	*74*	*99%*
	*No, blood sample should be available*	*1*	*1%*
If STIs are suspected, HIV screening should be performed	No	1	1%
	Yes	78	99%

## Data Availability

The data that support the findings of this study are available from the corresponding author upon reasonable request.

## Supplementary Materials

The following supporting information can be downloaded at: https://www.mdpi.com/article/10.3390/v17050667/s1.

## Abbreviations

AEEH	Spanish Association for the Study of the Liver
AEHVE	Alliance for the Elimination of Viral Hepatitis in Spain
GEHEP	Study Group of Viral Hepatitis
HAV	hepatitis A virus
HBV	hepatitis B virus
HCV	hepatitis C virus
HDV	hepatitis D virus
HIV	human immunodeficiency virus
NCH	National Catalogue of Hospitals
PCR	Polymerase chain reaction
POC	Point-of-care
SEIMC	Spanish Society of Infectious Diseases and Clinical Microbiology
SEPD	Spanish Society of Digestive Pathology
STI	sexually transmitted infections
WHO	World Health Organization
