# Supplementary material for: Comprehensive Diagnosis of Viral Hepatitis in Spain: Bases for Implementation
Source: Viruses. 2025 May 3;17(5):667. doi: 10.3390/v17050667 (PMC12116091; doi:10.3390/v17050667)
Supplement: Supplementary file 1 [file viruses-17-00667-s001.zip › viruses-3628579-supplementary.pdf]

SUPPLEMENTARY MATERIAL: COMPREHENSIVE DIAGNOSIS OF VIRAL HEPATITIS IN SPAIN: BASES FOR IMPLEMENTATION

SURVEY TO EVALUATE THE DEGREE OF IMPLEMENTATION OF THE RECOMMENDATIONS FOR COMPREHENSIVE DIAGNOSIS OF VIRAL HEPATITIS IN A SINGLE ANALYTICAL SAMPLE (1st SURVEY)

The objective of the project is to collect information from **BEFORE** the publication of the recommendations for the comprehensive diagnosis of viral hepatitis. If you agree to participate in the project, the estimated time for completion is 15–20 minutes.

This questionnaire consists of the following sections of questions:

1. Respondent and centre information.
2. Questions related to diagnosis of hepatitis B and D.
3. Questions related to availability of viral hepatitis reflex testing.
4. Other diagnostic recommendations.
5. General measures: *Point-of*-care.
6. Integration of screening programmes (implementation of viral hepatitis elimination programmes).
7. Result communication strategies.
8. Opinion questions.

CONSENT TO PARTICIPATE IN THE PROJECT

I agree to participate and complete the following questions in the questionnaire:

☐ Yes
☐ No

SECTION 1: RESPONDENT AND CENTRE INFORMATION.

1. Select your specialty:
   ☐ Microbiology
   ☐ Hepatology
   ☐ Gastroenterology
   ☐ Internal medicine
   ☐ Infectious diseases
2. Name of the centre: ……………………….
3. Name of the person responsible for completing the survey: ……………………….
4. Number of hospital beds
   ☐ Group 2: 200-500 beds
   ☐ Group 3: 501-1000 beds
   ☐ Group 4: More than 1000 beds
5. Reference population (inhabitants): ……………………….
6. Is the centre a university teaching hospital?
   ☐ Yes
   ☐ No

SECTION 2: QUESTIONS RELATED TO THE DIAGNOSIS OF HEPATITIS B AND HEPATITIS

7. Does your centre carry out HBsAg testing?
   ☐ Yes
   ☐ No

8. Does your centre carry out anti-HDV testing?
   ☐ Yes
   ☐ No

9. Does your centre perform HDV-RNA testing?
   ☐ Yes
   ☐ No

   If HDV-RNA testing is NOT performed at your centre, is it outsourced to other centres?
   ☐ Yes
   ☐ No

SECTION 3: QUESTIONS RELATED TO THE INTEGRATION OF REFLEX TESTING OF VIRAL HEPATITIS

Definitions:

- **HBV reflex testing:** Systematic determination of HBV-DNA in the same analytical sample in which HBsAg is first detected.
- **HDV reflex testing:** Systematic determination of antibodies against HDV (anti-HDV) in all patients in whom HBsAg is detected for the first time in the same sample.
- **Dual HBV-HDV reflex testing:** Systematic determination of antibodies against HDV in all patients in whom HBsAg is detected for the first time + systematic determination of HDV-RNA in all patients in whom anti-HDV is detected for the first time in the same sample.
- **HCV reflex testing:** Systematic determination of HCV infection by PCR in all patients in whom anti-HCV is detected for the first time in the same sample.

10. Is reflex testing performed at your centre?
    ☐ Yes
    ☐ No

11. In the answer is yes, indicate the type of reflex testing: (several answers can be marked)
    ☐ HBV reflex testing
    ☐ HDV reflex testing
    ☐ Dual HBV-HDV reflex testing
    ☐ HCV reflex testing

SECTION 4: OTHER DIAGNOSTIC RECOMMENDATIONS

HEPATITIS A

12. In your centre, is the presence of IgG or total hepatitis A virus antibodies determined in all patients with chronic hepatitis B?
    ☐ Yes
    ☐ No

13. If the determination is carried out, is it carried out in the same analytical sample?
    ☐ Yes

☐ No

14. In your centre, is the presence of IgG or total hepatitis A virus antibodies determined in all patients with chronic hepatitis C?

☐ Yes
☐ No

15. If the determination is carried out, is it carried out in the same analytical sample?

☐ Yes
☐ No

HUMAN IMMUNODEFICIENCY VIRUS (HIV)

16. In your centre, is the presence of antibodies against HIV determined in all patients with chronic hepatitis B?

☐ Yes
☐ No

17. If the determination is carried out, is it carried out in the same analytical sample?

☐ Yes
☐ No

18. In your centre, is the presence of antibodies against HIV determined in all patients with chronic hepatitis C?

☐ Yes
☐ No

19. If the determination is carried out, is it carried out in the same analytical sample?

☐ Yes
☐ No

SECTION 5: GENERAL MEASURES: POINT-OF-CARE.

Definition:

- **Point-of-Care tests: tests** performed at the site of patient care for rapid diagnosis and easy blood sample collection.

20. In your portfolio of services, do you have point-of-care (POC) testing?

☐ Yes
☐ No

21. If YES is available, what types of POC testing are performed? (you can check multiple answers)

☐ Rapid capillary blood antibody test
☐ Oraquick® HCV (capillary blood/saliva)
☐ GeneXpert® HCV
☐ Dry Drop
☐ Other (please specify): ……………………….

22. If available, in your centre, are the results of the POC tests supervised by the central microbiology laboratories?

☐ Yes
☐ No

23. If available, are the POC results registered into patients' medical records?

☐ Yes

☐ No

SECTION 6: INTEGRATION OF SCREENING PROGRAMMES (IMPLEMENTATION OF VIRAL HEPATITIS ELIMINATION PROGRAMMES).

24. Do you have a community or regional plan for the management/elimination of viral hepatitis?

☐ Yes

☐ No

25. Is systematic screening performed in addiction centres?

☐ Yes

☐ No

26. What type of screening is performed in previously treated patients (in patients at risk of reinfection)? (you can select multiple answers)

☐ Conventional extraction

☐ Dry Drop

☐ GeneXpert®

SECTION 7: RESULT COMMUNICATION STRATEGIES.

27. In your centre, do you have any kind of system for alerting the doctor responsible for the treatment of these patients to the existence of active viral hepatitis?

☐ Yes

☐ No

28. If YES, for which types of hepatitis do you provide alerts? (you can select multiple answers)

☐ HBV

☐ HDV

☐ HCV

29. Does your centre have an automated system for scheduling an appointment with a specialist for patients with positive serology for any viral hepatitis?

☐ Yes

☐ No

30. If YES, is this alert communicated to the service in charge of managing the appointment?

☐ Yes

☐ No

SECTION 8: OPINION QUESTIONS.

31. Do you think that the test for HAV should be performed together with tests for viral hepatitis B, C and D in the same blood sample in all patients with a positive result for HBV or HCV?

☐ Yes

☐ No

32. If no, what is the reason?

………

33. Do you think that HIV antibody serology should be performed together with viral hepatitis B, C and D testing in the same blood sample for all patients?

☐ Yes

☐ No

34. If no, what is the reason?

………………………………………………………………………………………………………

35. Do you think that comprehensive diagnosis of viral hepatitis should be performed in patients with suspected sexually transmitted infections—STIs (syphilis, gonorrhoea, chlamydia, herpes)?

☐ Yes

☐ No

36. If you think yes, should a blood sample be available for all patients with a suspected STI diagnosis?

☐ Yes

☐ No

37. Do you think that screening for HIV should be performed for patients with suspected sexually transmitted diseases—STIs (syphilis, gonorrhoea, chlamydia, herpes)?

☐ Yes

☐ No

Task force Spanish Group for comprehensive hepatitis diagnosis (alphabetic order*)

Principal Researcher		Hospital	Province
Antonio	Aguilera Guirao	C. H. Universitario de Santiago de Compostela	A Coruña
María Jesús	Alcaraz Soriano	H. Clínico Universitario de Valencia	Valencia
Roberto	Alonso Fernández	H. General Universitario Gregorio Marañón	Madrid
Maitane	Aranzamendi Zaldumbide	H. Universitario de Donostia	Guipúzcoa
Marta	Arias Temprano	H. Universitario de Cabueñes	Asturias
Ana	Arribi Vilela	H. Clínico San Carlos	Madrid
Teresa	Arroyo Serrano	H. Universitario Príncipe de Asturias	Madrid
Raquel	Baluja Pino	H. Ribera Povisa	Pontevedra
Rafael	Benito Ruesca	H. Clinico Universitario Lozano Blesa	Zaragoza
Samuel	Bernal Martínez	C.H. de Especialidades Virgen de Valme	Sevilla
Miriam	Blasco Alberdi	H. Universitario San Pedro	La Rioja
Ana	Blázquez Abellán	H. General Universitario Santa Lucia	Murcia
María Eliecer	Cano García	H. Universitario Marqués de Valdecilla	Cantabria
Purificación	Cantudo Muñoz	H. Comarcal San Agustín	Jaén
Laura	Cardeñoso Domingo	H. Universitario de la Princesa	Madrid
Ángel	Castaño Nuñez	H. Universitario Fundación Jiménez Díaz	Madrid
Encarnación	Clavijo Frutos	H. Universitario Virgen de la Victoria	Málaga
Rodolfo	Copado Carretero	H. Dr. José Molina Orosa	Lanzarote
Sandra	Cortizo Vidal	C. H. Universitario de Vigo	Pontevedra
Alberto	de la Iglesia Salgado	H. Comarcal Infanta Elena	Huelva
Jose Luis	Díaz de Tuesta del Arco	H. Universitario Basurto	Vizcaya
Felícitas	Díaz-Flores Estévez	C. H. Universitario de Canarias	Tenerife
Victoria	Dominguez-Márquez	H. Arnau de Vilanova de Valencia	Valencia
José María	Eiros Bouza	H. Universitario Río Hortega	Valladolid
Victoria	Fernández Baca	H. Universitario Son Llatzer	Mallorca
Felipe Manuel	Fernández Cuenca	H. Universitario Virgen de la Macarena	Sevilla
Isabel	Fernández Natal	C. Asistencial Universitario de León	León

Task force Spanish Group for comprehensive hepatitis diagnosis (alphabetic order*)

Principal Researcher		Hospital	Province
Gema	Fernández Rivas	H. Universitario Germans Trias i Pujol	Barcelona
Fernando	Fernández Sánchez	H. Universitario Costa del sol	Málaga
Carolina	Freyre Carrillo	H. Universitario Puerto Real	Cádiz
Juan Carlos	Galán Montemayor	H. Universitario Ramón y Cajal	Madrid
Fernando	García García	H. Universitario San Cecilio	Granada
Sonsoles	Garcinuño Pérez	H. Clinico Universitario de Valladolid	Valladolid
Carmen	Gómez González	H. Universitario de Álava	Álava
Alejandro	Gonzalez Praetorius	H. Universitario de Guadalajara	Guadalajara
María José	Gude González	C. H. Universitario de Lugo (H. Lucus Augusti)	Lugo
Maria Araceli	Hernández Betancor	H. Universitario Insular de Gran Canaria	Las Palmas
Juan Carlos	Hurtado Negreiros	H. Clinic de Barcelona	Barcelona
María Asunción	Iborra Bendicho	H. Clínico Universitario Virgen de la Arrixaca	Murcia
María Magdalena	Lara Pérez	H. Universitario Ntra Sra de Candelaria	Tenerife
María Josefa	López de Goicoechea	H. Galdakao-Usansolo	Vizcaya
María Fátima	López Fabal	H. Universitario de Móstoles	Madrid
Jose Luis	López Hontangas	H. Universitario y Politécnico La Fe	Valencia
María del Carmen	Lozano Domínguez	H. Universitario Virgen del Rocío	Sevilla
María Pilar	Luzón García	H. Universitario Poniente	Almería
María Dolores	Maciá Romero	H. Universitario Son Espases	Mallorca
Olalla	Martinez Macias	H. Universitario de La Ribera	Valencia
Ana	Miqueleiz Zapatero	H. Universitario de Navarra	Navarra
Luz	Moldes Suárez	C. H. Universitario de A Coruña	A Coruña
Laura	Molina Esteban	H. Universitario Fuenlabrada	Madrid
Dolores	Montero Vega	H. Universitario La Paz	Madrid
Natalia	Montiel Quezel-Guerraz	H. Universitario Puerta del Mar	Cádiz
Carmen	Muñoz Almagro	H. de Sant Joan de Déu	Barcelona
María	Navarro Aguirre	H. Universitari de Vic	Barcelona

Task force Spanish Group for comprehensive hepatitis diagnosis (alphabetic order*)

Principal Researcher		Hospital	Province
María Dolores	Navarro Martínez	H. General Universitario Morales Meseguer	Murcia
María Dolores	Ocete Mochón	H. General Universitario de Valencia	Valencia
Valle	Odero Bernal	H. Universitario de Jérez de la Frontera	Cádiz
Patricia	Ordoñez Barrosa	C. H. Universitario de Ferrol	A Coruña
Nieves	Orta Mira	H. Comarcal Francesc de Borja	Valencia
Diego	Ortega Larrea	H. Universitario Miguel Servet	Zaragoza
María José	Pena López	H. Universitario de Gran Canaria Dr. Negrín	Las Palmas
Ana Belén	Pérez Jiménez	H. Universitario Reina Sofía	Córdoba
J. Alfredo	Pérez Rivilla	H. Universitario 12 de octubre	Madrid
Andrés	Quesada	H. Comarcal San Juan de la Cruz	Jaén
Gabriel	Reina González	Clínica Universidad de Navarra	Navarra
Juan Carlos	Rodríguez Díaz	H. General Universitario Dr Balmis	Alicante
Francisco	Rodríguez Frías	H. Universitario Vall d'Hebron	Barcelona
Manuel Ángel	Rodríguez Maresca	C. H. de Especialidades Torrecárdenas	Almería
Luis	Rodríguez Otero	C. H. y Universitario de Ourense	Ourense
Salud	Rodríguez Pallares	H. San Pedro Alcántara	Cáceres
María Mercedes	Rodríguez Pérez	H. Universitario Central de Asturias	Asturias
María	Rodríguez Velasco	H. El Bierzo	León
Susana	Sabater Vidal	H. General Universitario de Castellón	Castellón
Ruth	Sáez de la Maleta Úbeda	H. Universitario de Burgos	Burgos
Mónica	Sánchez Oñoro	H. General Fuerteventura	Las Palmas
Gemma María	Sierra Dorado	H. Universitario San Agustín	Asturias
Raquel	Téllez Pérez	H. Universitario Fundación Jiménez Díaz	Madrid
Luis	Torres Sopena	H. General San Jorge	Huesca
Matilde	Trigo Daporta	C. H. Universitario Pontevedra	Pontevedra
María Isabel	Zamora Cintas	H. Central de la Defensa Gómez Ulla	Madrid
